# Compatibilization of Poly(Lactic Acid) (PLA) and Bio-Based Ethylene-Propylene-Diene-Rubber (EPDM) via Reactive Extrusion with Different Coagents

**DOI:** 10.3390/polym12030605

**Published:** 2020-03-06

**Authors:** Alexander Piontek, Oscar Vernaez, Stephan Kabasci

**Affiliations:** 1 Department of Bio-based Plastics, Fraunhofer UMSICHT, Institute for Environmental, Safety and Energy Technology, Osterfelder Str. 3, 46047 Oberhausen, Germany; 2 Department of Research and Development in Products and Applications, Neste Corporation, PBO 310, FI-06101 Porvoo, Finland

**Keywords:** PLA, EPDM, soybean oil, bio-based polymers, reactive extrusion, compatibilization, dynamic vulcanization, thermoplastic vulcanizate

## Abstract

Much effort has been made to enhance the toughness of poly (lactic acid) (PLA) to broaden its possible range of usage in technical applications. In this work, the compatibility of PLA with a partly bio-based ethylene-propylene-diene-rubber (EPDM) through reactive extrusion was investigated. The concentration of EPDM in the PLA matrix was in the range of up to 20%. The reactive extrusion was carried out in a conventional twin-screw extruder. Contact angle measurements were performed to calculate the interfacial tension and thus the compatibility between the phases. The thermal and mechanical properties as well as the phase morphology of the blends were characterized. A copolymer of poly (ethylene-co-methyl acrylate-co-glycidyl methacrylate) (EMAGMA) was used as compatibilizer, which leads to a significant reduction in the particle size of the dispersed rubber phase when compared with the blends without this copolymer. The use of EMAGMA combined with soybean oil (SBO) and a radical initiator enhances the elongation at break of the compound. The results indicate that the reduction of the particle size of the dispersed phase obtained with the compatibilizer alone is not sufficient to improve the mechanical properties of the blend system. The induced radical reactions also influenced the mechanical properties of the blend significantly.

## 1. Introduction

Poly (lactic acid) (PLA) is a commercially available bio-based and biodegradable polyester which exhibits good mechanical properties such as high tensile modulus and strength. These properties make it suitable to substitute conventional polymers in different industrial applications. Nevertheless, the use of PLA in technical applications is limited due to its inherent brittleness and low elasticity. In the past years, much effort has been made to overcome these disadvantages and to improve the thermo-mechanical properties of PLA via different solution approaches [1,2].

One possible way to improve the mechanical properties of PLA is the physical blending with low molecular weight additives or plasticizers, e.g., derivatives of citric acid [3] or glycerol [4]. This improves the ductility as well as the processability, but due to their high volatility, these small molecules tend to evaporate during processing and to migrate to the polymer surface. This can be avoided by using oligomeric or polymeric blending partners. Burgos et al. used oligomeric lactic acid for plasticization [5]. This increased the elasticity but on the other hand greatly decreased the tensile strength and Young’s modulus. Similar results were found when Jacobsen and Fritz used oligomeric poly (ethylene glycol) (PEG), a partial fatty acid ester and a glucose monoester as plasticizers. Only PEG contributed to an additional increase in impact resistance in contrast to the other additives [6,7]. Blends with oligomeric poly (1,2-propylene glycol adipate) (PPA) prepared by Zhang et al. showed similar behavior but also with the same disadvantages [7]. Many publications are available describing that PLA was physically blended with different other polymers, such as poly(methyl methacrylate) (PMMA) [8], poly(butylene succinate) (PBS) [9], poly(ethylene-co-butyl acrylate) (EBA) [10], poly(ethylene-co-octylene) (POE) [11], thermoplastic polyurethane (TPU) [12]. Blending of PLA with other polymers is limited due to incompatibility and the resulting phase separation. Therefore, different compatibilizers were used in the aforementioned studies to decrease interfacial tension and thus enhance the mechanical properties. However, the synthesis of tailor-made compatibilizers can be rather time consuming or expensive, while there are only few commercially available copolymers that can be used as specialty compatibilizers for PLA blend systems.

To overcome the disadvantages of physical blending, reactive compatibilization has attracted much attention in recent years. This general description covers different approaches. One possibility is the use of small multi-functional reactive components, which can react with both phases of the polymer blend. Another example is the use of diisocyanates such as 4,4′-methylene diphenyl diisocyanate (4,4′-MDI) or epoxy group containing copolymers such as styrene–acrylate–glycidyl methacrylate copolymer (Joncryl^®^), which can react with hydroxyl, carboxyl, or amine groups of different polymer types [13,14]. In both cases, the compatibilizer covalently attaches to both phases and acts as a chemical link in the interface. An alternative method is the in situ production of physical compatibilizers in polyester–polyester blends, which can be achieved by the use of transesterification catalysts [15]. It is also possible to use compatibilizers, which are compatible with either matrix or dispersed phase and can react with the other phase. Common reactive groups are isocyanates, epoxides, or anhydrides, depending on the polymer blend in which they are to be used [13,14].

A special subcategory of reactive compatibilization is the use of peroxides in polymer blends. Peroxides can induce radical reactions in both polymer phases of a blend. Due to the termination reactions that happen in the interfacial region, copolymers of both of the polymer phases are created which can then act as physical compatibilizers. Furthermore, the radical reactions have an influence on the properties of the polymer phases themselves. When using cross-linkable polymers so called thermoplastic vulcanizates (TPV) are obtained, which is why this kind of reactive compatibilization is also called dynamic vulcanization. TPVs are polymer blends that consist of a thermoplastic matrix with a cross-linked and elastic dispersed phase. The properties of TPVs range from impact modified thermoplastics to ductile thermoplastic elastomers that combine the elastic properties of elastomers with the (re-)processability of thermoplastics [16]. There are many publications describing dynamically vulcanized biopolymer blends. Most of them use PLA as matrix phase with different commercially available soft segments like natural rubber (NR) [17,18], epoxidized natural rubber (ENR) [19,20], poly(ethylene-co-vinyl acetate) (EVA) [21,22] and different synthesized bio-based unsaturated elastomers [23,24]. Nevertheless, there are also publications with other matrices, for example polyhydroxyalkanoates (PHAs) [25,26]. Most of the mentioned publications use a batch process to produce the bio-based TPVs, e.g., by using an internal mixer and long reaction times.

In this study, we investigated the continuous production of PLA-based TPVs by reactive extrusion. For the dispersed phase, we used a commercially available and partially bio-based ethylene propylene diene monomer rubber (EPDM). Viscosity and reactivity of the soft phase was varied by using soybean oil as a bio-based and reactive plasticizer. A reactive compatibilizer is used to further enhance the compatibility of the different phases during reactive extrusion.

## 2. Materials and Methods

### 2.1. Materials

Poly(lactic acid) (PLA, Ingeo^™^ 4044D) was purchased from Natureworks LLC, Minnetonka, MN, USA, with a melt flow index (MFI) of 6 g per 10 min (210 °C, 2.16 kg) and an L-lactide content of about 96%. Ethylene propylene diene monomer rubber (EPDM, Keltan^®^ Eco 8550) was kindly provided by Arlanxeo Netherlands B.V., Geleen, NL, with a mooney viscosity of 80 MU (ML(1 + 4), 125 °C), an ethylene content of 55% and an ethylidene norbonene content of 5.5%. Poly(ethylene-co-methyl acrylate-co-glycidyl methacrylate) (EMAGMA, Lotader^®^ AX8900) was purchased from Arkema S.A., Colombes, FR, with a methyl acrylate content of 24%, a glycidyl methacrylate content of 8% and a MFI of 6 g per 10 min (190 °C, 2.16 kg). Tert-butylperoxy 2-ethylhexyl carbonate (TBEC, Trigonox^®^ 117) was purchased from Akzo Nobel Nederland BV, Arnhem, NL, as a PLA masterbatch with a peroxide content of 40%. Soybean oil (SBO, refined, extra pure) was purchased from Carl Roth GmbH & Co. KG, Karlsruhe, DE. Calcium carbonate (Omyalite^®^ 90 OM), was purchased from Omya International AG, Oftringen-Aargau, CH. Dimethyl sulfoxide (DMSO), ethylene glycol and thiodiglycol were purchased from Merck KGaA, Darmstadt, DE. We used all materials as received.

### 2.2. Sample Preparation

EPDM was cut into pieces and granulated under liquid nitrogen in a cutting mill. The granulate was powdered with 2.5 wt % of calcium carbonate to prevent agglomeration. Prior to blending, PLA was dried overnight at 60 °C in a dry-air drier. Blends with varying ratios of the different components were prepared in an intermeshing, co-rotating twin-screw extruder (ZSK 25 from Coperion GmbH, Stuttgart, DE, screw diameter 25 mm, L/D ratio 40), at a screw speed of 250 rpm and a mass flow rate of 12 kg/h. The temperature was set to 190 °C for each zone (except for first and second zone with 60 °C and 170 °C, respectively). The strand was cooled in a water bath, pelletized via strand granulation and dried in a dry-air dryer at 60 °C. The granulates were compression molded to 2 mm thick sheets for tensile tests and to 4 mm thick sheets for impact fracture tests in a Scientific LP-S-20 compression molding machine from LabTech Engineering Co. LTD, Samutprakarn, TH, at 200 °C and 75 kN for 5 min. The sheets were milled to rectangular bars (130 × 10 × 2 mm for tensile tests and 80 × 10 × 4 mm for impact fracture tests) in a milling machine.

The samples were prepared with varying ratios of the different components, which is represented by their nomenclature. Samples contained different amounts of the following components with their corresponding abbreviation in brackets used in the sample nomenclature: PLA (P), EPDM (E), SBO (S), EMAGMA (C), and TBEC (T). The number following the abbreviation corresponds to its amount in mass parts (pph). For example the sample P80-E16-S4-C5-T0.2 consists of 80 parts PLA, 16 parts EPDM, 4 parts SBO, 5 parts EMAGMA, and 0.2 parts TBEC. PLA, EPDM and SBO were always calculated to give 100 parts whereas EMAGMA and TBEC were added on top. The amount of TBEC is also given in parts per hundred rubber (phr) corresponding to the total amount of soft phase (EPDM and SBO).

### 2.3. Characterization

#### 2.3.1. Surface and Interfacial Properties

To quantify the differences in polarity of the materials used in this study, the corresponding surface free energy (SFE) was determined. The SFE of a liquid or solid and its polar and dispersive parts was calculated via contact angle measurements. The relation between the contact angle and the SFE of the different phases is described by the Young Equation (1):

(1) $$\sigma_{s}\;=\;\sigma_{sl}+\sigma_{l}\cdot \cos\theta ,$$

where $\sigma_{s}$ is the SFE of the solid, i.e., the polymeric sample, $\sigma_{l}$ the SFE of the liquid, $\sigma_{sl}$ the interfacial tension between the solid and the liquid and θ the contact angle. According to the method from Owens–Wendt–Rabel–Kaelbe [27,28,29] built on the theory of Fowkes, Equation (2) is used for calculating the interfacial tension by using the geometric mean:

(2) $$\sigma_{ij}\;=\;\sigma_{i}+\sigma_{j}-2\sqrt{\sigma_{i}^{d}\cdot \sigma_{j}^{d}}-2\sqrt{\sigma_{i}^{p}\cdot \sigma_{j}^{p}},$$

where $\sigma^{d}$ and $\sigma^{p}$ are the dispersive and polar parts of the SFE, respectively. Contact angle measurements were performed with several test liquids with known $\sigma^{d}$ and $\sigma^{p}$ values as listed in Table 1

A drop with about 3–5 µL of the test liquid was placed on a compression molded plate under test conditions (23 °C, 50% relative humidity). The drop shapes were recorded using a VHX 1000 digital microscope from Keyence Deutschland GmbH, Neu-Isenburg, DE, and analyzed using ImageJ open source software with the contact angle plugin developed by M. Brugnara [33]. All results are presented as the average of at least five measurements per sample and test liquid.

The interfacial tensions between the different polymeric phases were calculated according to Equation (2) and in comparison according to Wu’s model [34] using the harmonic mean, Equation (3):

(3) $$\sigma_{ij}\;=\;\sigma_{i}+\sigma_{j}-4\left(\frac{\sigma_{i}^{d}\cdot \sigma_{j}^{d}}{\sigma_{i}^{d}+\sigma_{j}^{d}}+\frac{\sigma_{i}^{p}\cdot \sigma_{j}^{p}}{\sigma_{i}^{p}+\sigma_{j}^{p}}\right).$$

With the calculated interfacial tensions, the spreading coefficient S and the wetting coefficient ω are calculated according to Equations (4) and (5) [35,36]:

(4) $$S\;=\;\sigma_{AB}-\sigma_{BC}-\sigma_{AC},$$

(5) $$\omega \;=\;\frac{\sigma_{BC}-\sigma_{AC}}{\sigma_{AB}},$$

where $\sigma_{AB}$ is the interfacial tension between the matrix A (PLA) and the dispersed phase B (EPDM), $\sigma_{BC}$ the interfacial tension between EPDM and the compatibilizer C (EMAGMA), and $\sigma_{AC}$ the interfacial tension between PLA and EMAGMA.

#### 2.3.2. Morphological Properties

For a qualitative analysis of compatibilization, a Molau test was performed [37,38]. For this, the samples were dissolved in dichloromethane (DCM) (5% *w/v*) under stirring. The obtained suspensions were characterized visually and the degree of turbidity was taken as an indicator for a successful compatibilization. Scanning electron microscopy (SEM) was performed with a Vega 3 from TESCAN GmbH, Dortmund, DE, with 20 kV acceleration voltage. The compression molded specimens were submerged in liquid nitrogen for 10 min and quickly sliced with a Leica RM 2265 microtome. Additionally, SEM images were taken from selected tensile fracture surfaces. To prevent electrostatic charging, the samples were sputter coated with gold under vacuum prior to observation. Analysis of the SEM pictures was performed with open source ImageJ software. To quantify the phase morphology a statistical analysis of the particle sizes in the blend was performed. From at least three different locations on the sample surface at least 200 particles were chosen—unless otherwise stated—and the according diameters were calculated assuming circular particles. The particle size distribution parameter σ and average particle size d were calculated with the following equations.

(6) $$\ln d\;=\;\frac{\sum_{i\;=\;1}^{N}n_{i}\ln d_{i}}{\sum_{i\;=\;1}^{N}n_{i}},$$

(7) $$\ln\sigma \;=\;\sqrt{\frac{\sum_{i\;=\;1}^{N}n_{i}{\left(\ln d_{i}-\ln d\right)}^{2}}{\sum_{i\;=\;1}^{N}n_{i}}},$$

where $n_{i}$ is the number of particles with diameter $d_{i}$ and σ is a parameter describing the particle size distribution [39]. These values were used for the calculation of the inter particle distance L according to the model from Wu (Equation (8)) [40]:

(8) $$L\;=\;d\left[{\left(\frac{\pi }{\phi }\right)}^{\frac{1}{3}}\exp\left(1.5\ln^{2}\sigma \right)-\exp\left(0.5\ln^{2}\sigma \right)\right],$$

where ϕ is the volume fraction of the dispersed phase.

#### 2.3.3. Mechanical Properties

Tensile tests were performed with a 5567A universal testing system from Instron GmbH, Darmstadt, Germany, at a speed of 50 mm/min in accordance to DIN EN ISO 527-1. Testing speed for the determination of the Young’s modulus between 0.05 and 0.25% elongation was 1 mm/min. Charpy impact fracture tests were performed with a Ceast 9050 pendulum impact testing machine from Instron GmbH, Darmstadt, Germany, with notched (type A) specimens using a 5 J instrumented pendulum according to DIN EN ISO 179-2. Test specimens were stored under test conditions (23 °C, 50% relative humidity) for at least 16 h prior to testing. All results are presented as the average from five measurements. Graphs of tensile tests represent the median measurement with regard to elongation at break.

#### 2.3.4. Thermal Properties

Differential scanning calorimetry (DSC) was performed with a DSC 204 F1 Phoenix from Erich NETZSCH GmbH & Co. Holding KG, Selb, Germany, equipped with a liquid nitrogen cooling system. The device is regularly calibrated using an indium standard. The samples were cooled to −100 °C, heated up to 230 °C, cooled again to −100 °C and heated up to 250 °C with heating and cooling rates of 10 K/min under nitrogen atmosphere.

## 3. Results and Discussion

### 3.1. Surface and Interfacial Properties

In this work, we used the compatibilizer EMAGMA to improve the compatibility between the PLA and EPDM phases in a blend. To quantify the polarity and thus predict the compatibility of PLA, EPDM and EMAGMA their interfacial tensions were measured. The values were calculated from contact angle measurements with different test liquids. Results are shown in Table 2.

The results indicate that the overall SFE of the compatibilizer EMAGMA lies between those of PLA and EPDM. As a first approximation, these results show that EMAGMA can be a suitable physical compatibilizer for this polymer blend. To make a more accurate statement, the interfacial tensions and the resulting values for the spreading and wetting coefficients were calculated according to Equations (2)–(5). The results are shown in Table 3.

It is to be noted that there are differences in the results whether using the harmonic or geometric mean for the calculation. Wetting of EMAGMA on the dispersed phase occurs for S>0. Considering the wetting coefficient ω a more accurate statement is possible. For ω>1 EMAGMA is mainly located in the PLA phase, for ω<−1 EMAGMA is mainly located in the EPDM phase and for −1 <ω<1 EMAGMA is mainly located in the interphase [41]. From the results it can be observed that the spreading coefficient for the spreading of EMAGMA on EPDM is >0. This indicates that EMAGMA can spread on the EPDM phase surface inside the blend.

The wetting coefficient also confirms this. As the value lies between −1 and 1, EMAGMA should mainly be located in the interfacial area and not inside one of the two phases. According to these results, EMAGMA is well suited as a physical compatibilizer for PLA and EPDM.

**Figure 1:**
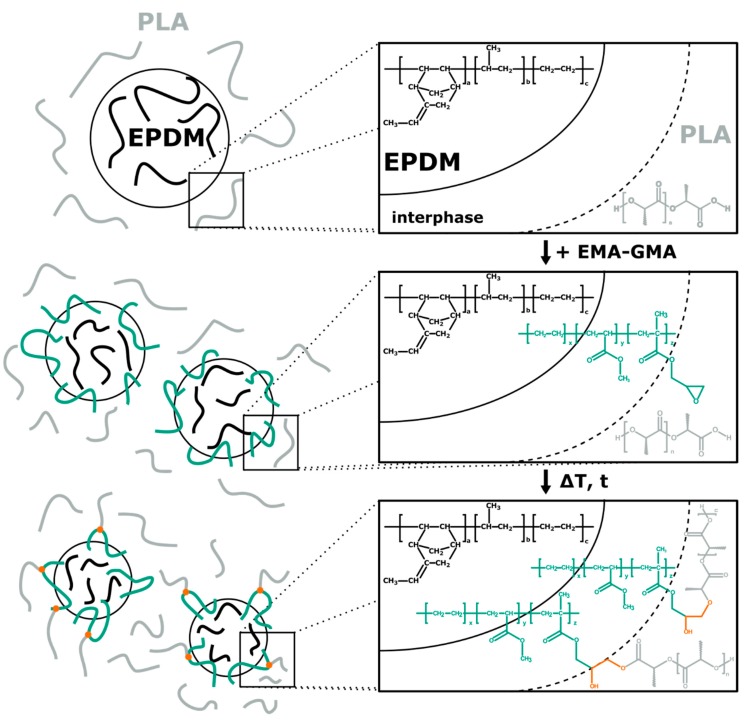
Schematic representation of physical compatibilization of PLA and EPDM with EMAGMA and possible chemical reactions.

In addition to the physical compatibilization effects of EMAGMA, it contains epoxy groups that can react with hydroxyl or carboxyl groups of the PLA [42,43]. These reactions would lead to copolymers of PLA and EMAGMA, which would have a higher compatibilizing effect than the pure EMAGMA. This is illustrated in Figure 1.

### 3.2. Morphological Properties

**Figure 2:**
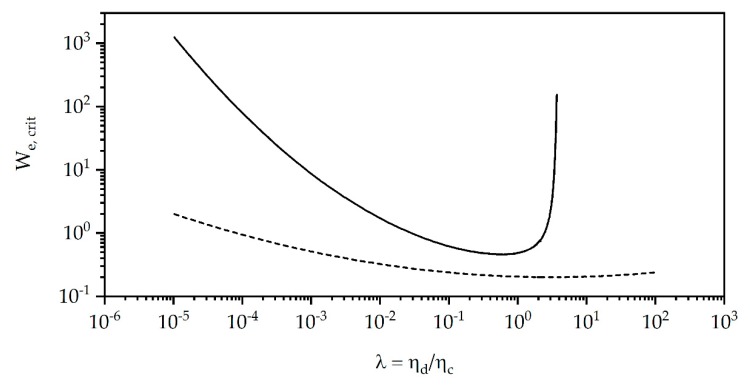
Critical Weber number $W_{e,\;crit.}$ for shear flow (solid line) and elongational flow (dotted line) as a function of the viscosity ratio λ of dispersed phase $\eta_{d}$ to continuous phase $\eta_{c}$ from empirical equations according to de Bruijn [45] and Peters et al. [46].

One effect of good compatibilization in polymer blends is a decrease in particle size of the dispersed phase, i.e., in this study the EPDM. During processing, there is an equilibrium between droplet breakup due to deformation by shear stress and coalescence of droplets by collision. One factor describing the droplet breakup is the Weber number $W_{e}$, often found as capillary number $C_{a}$, which is a dimensionless number defined as the ratio of deforming inertial forces to cohesive forces acting on the droplets, as presented in Equation (9),

(9) $$W_{e}\;=\;\frac{\dot{\gamma }\cdot d_{0}\cdot \eta_{c}}{2\cdot \sigma },$$

where $\dot{\gamma }$ is the shear rate, $d_{0}$ the droplet average diameter, $\eta_{c}$ the matrix viscosity, and σ the interfacial tension between the matrix and dispersed phase. During extrusion, the Weber number has to exceed a critical value $W_{e,\;crit.}$ for the droplet to break up in the shear flow, otherwise, no further breakup will occur. The critical Weber number was empirically studied by different working groups [44] and depends highly on the viscosity ratio of the two polymer phases, which is illustrated in Figure 2.

During processing the value of the Weber number changes caused by various parameter changes during the reactive extrusion. The main mechanism is the droplet breakup during processing. This decreases $d_{0}$ in Equation (9) and leads to a smaller value of the Weber number. As a consequence, the size of the dispersed soft phase particles reaches a minimum during processing at constant processing conditions, i.e., constant shear rate and matrix viscosity, when $W_{e}\leq W_{e,crit}$. Compatibilization of the different phases, i.e., decreasing the interfacial tension σ, increases the initial value of $W_{e}$ which leads to smaller particles. Influencing the viscosities of either phase affects the initial viscosity ratio λ and therefore the necessary $W_{e,crit}$ for droplet breakup. Adding a plasticizer influences the viscosity ratio λ by a constant factor, whereas dynamical crosslinking in any phase results in a time dependent change of λ.

**Figure 3:**
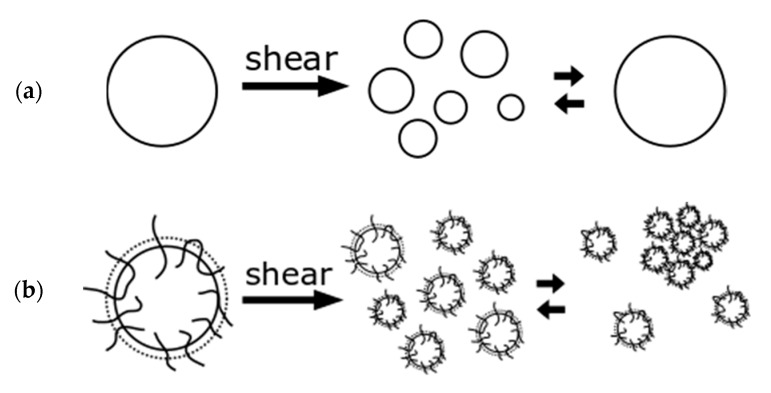
(**a**) Coalescence of dispersed particles without compatibilizer and (**b**) inhibition of coalescence with compatibilizer and possible agglomeration.

Furthermore, compatibilizers also reduce the probability of coalescence and hence maintain smaller particle sizes of the dispersed phase, as illustrated in Figure 3. The same mechanism is also observed when the dispersed phase consists of cross-linked particles. Because we used a combination of compatibilization and dynamic vulcanization of the dispersed phase, we expected the best results when combining both mechanisms.

**Figure 4:**
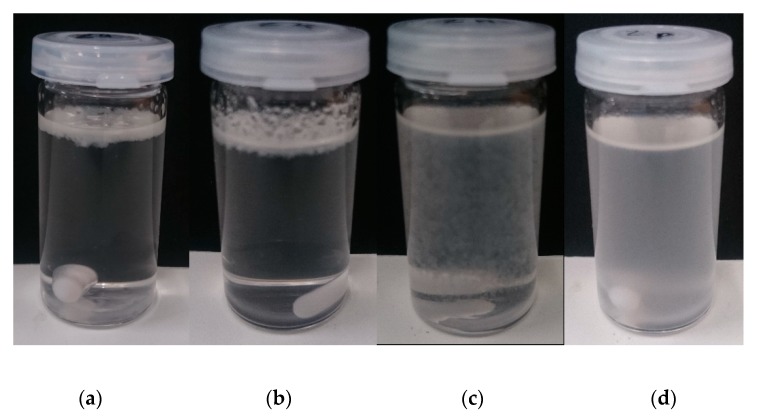
Samples dissolved in dichloromethane (DCM) directly after stirring. (**a**) PLA/EPDM/SBO, (**b**) PLA/EPDM/SBO/TBEC, (**c**) PLA/EPDM/SBO/EMAGMA, and (**d**) PLA/EPDM/SBO/EMAGMA/TBEC.

To gain a qualitative statement of the compatibilization, a Molau test was performed. The dissolved samples were photographed directly after stirring. The results are shown in Figure 4.

PLA as well as SBO are dissolved in dichloromethane (DCM) while EPDM remains as a solid dispersed phase that directly starts to separate due to differences in density compared to DCM (Figure 4a). The same can also be observed when TBEC is present (Figure 4b). This indicates that there is no compatibilization between PLA and the plasticized EPDM-SBO phase through radical interactions at the used processing conditions. Besides its plasticizing effect, the SBO was supposed to act as a reactive plasticizer due to reactions of the double bonds of the unsaturated fatty acid chains with the EPDM during vulcanization. As depicted by the results of the Molau test, the use of SBO did not lead to a reaction between the two polymer phases. When adding EMAGMA to the blend, a flocculation and dispersion of the particles can be observed (Figure 4c), which indicates an increased compatibility between the two phases. This corresponds to the expectations from the results of the interfacial tension calculations. The dispersion of the blend containing all of the additives, i.e., SBO, EMAGMA, and TBEC, is more homogenous compared to the dispersion without TBEC (Figure 4d), indicating an even higher degree of compatibility.

**Figure 5:**
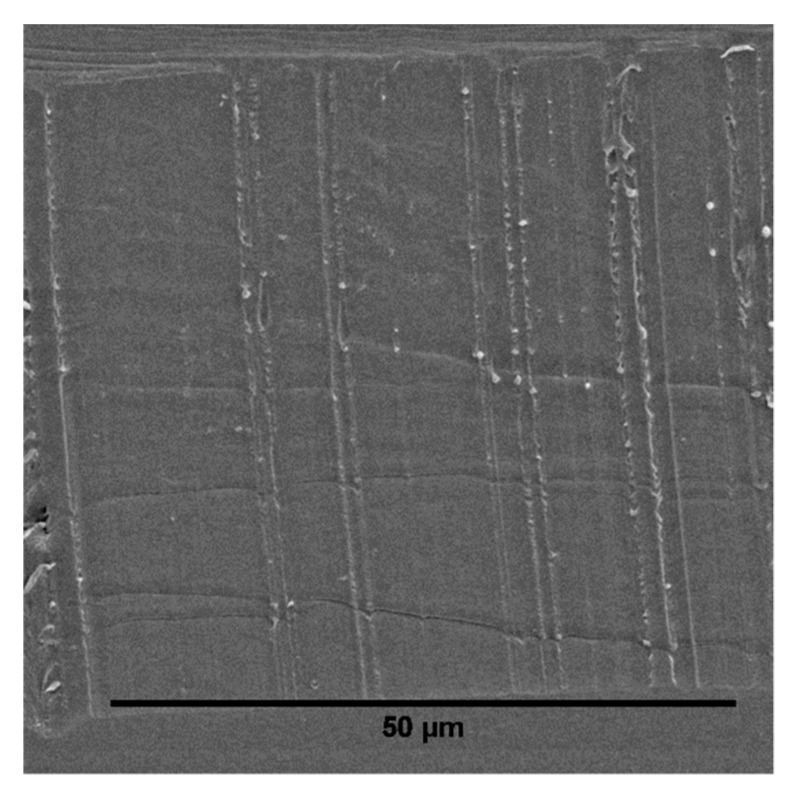
Cryo-sliced surface of pure PLA.

**Figure 6:**
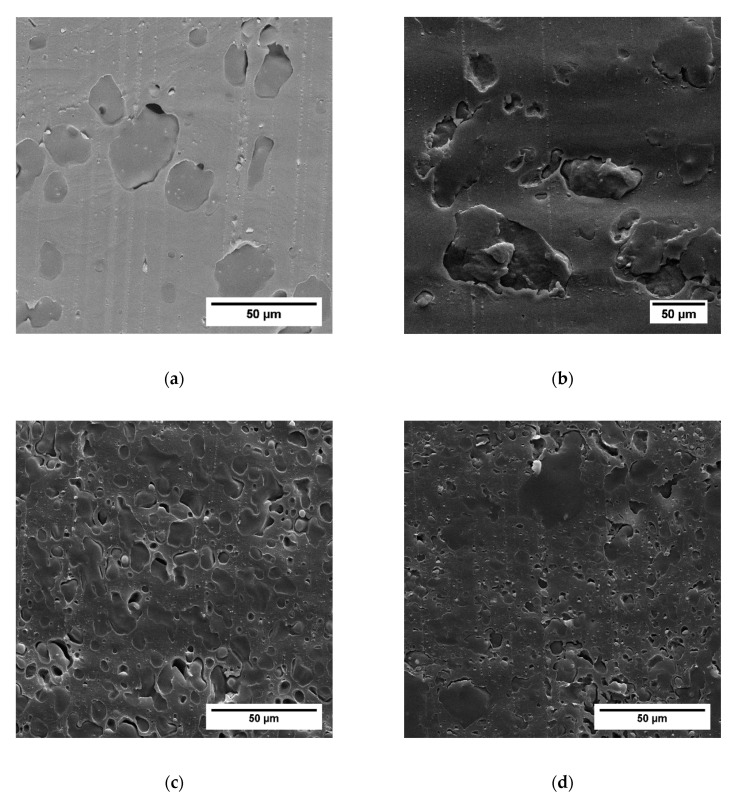
Cryo-sliced surfaces of the samples without soybean oil (SBO). (**a**) Without EMAGMA and Tert-butylperoxy 2-ethylhexyl carbonate (TBEC), (**b**) with 1 phr TBEC, (**c**) with 5 pph EMAGMA, and (**d**) with 5 pph EMAGMA and 1 phr TBEC.

**Figure 7:**
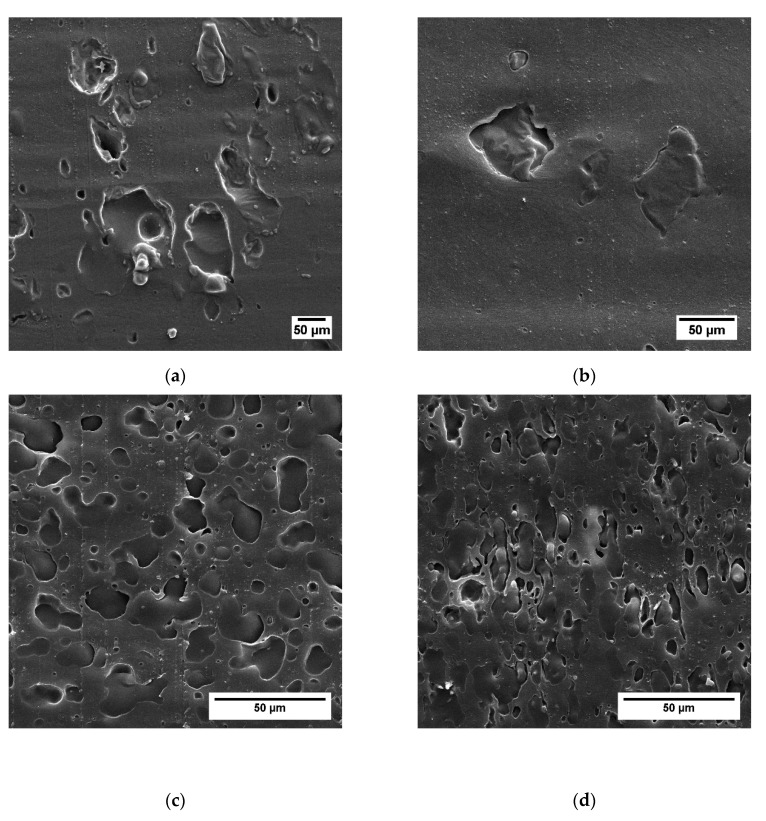
Cryo-sliced surfaces of the blends with 20 wt % SBO inside the soft phase. (**a**) Without EMAGMA and TBEC, (**b**) with 1 phr TBEC, (**c**) with 5 pph EMAGMA, and (**d**) with 5 pph EMAGMA and 1 phr TBEC.

For a more detailed analysis of the phase morphology of the prepared blends, we performed SEM measurements of sliced sample surfaces, which are depicted in Figure 5, Figure 6 and Figure 7. Cryo-slicing of the pure PLA shows cutting grooves, but no second phase. On the other pictures, it is possible to differentiate between those grooves and the elastomeric soft phase when analyzing the samples regarding the size and distribution of the soft phase. We can see qualitatively in Figure 6 and Figure 7 that the addition of TBEC alone did not contribute to the size reduction of the EPDM particles. Only the addition of EMAGMA as a compatibilizer reduces the particle size of the soft phase significantly. This is in accordance to the mechanism of droplet breakup considering Equation (9). The combination of EMAGMA and TBEC does not have an additional effect on the size of the soft phase.

The SEM results also show that there is no qualitative improvement of the interfacial adhesion through the compatibilization or the dynamic vulcanization. We still can see voids between PLA and EPDM and to some extent deformed EPDM particles inside these holes. These effects should not occur if the interfacial adhesion was significantly improved. This lack of adhesion also plays an important role in the mechanical properties as the stress cannot be transferred from the matrix to the soft phase and the formation of voids will be the main mechanism during deformation.

**Figure 8:**
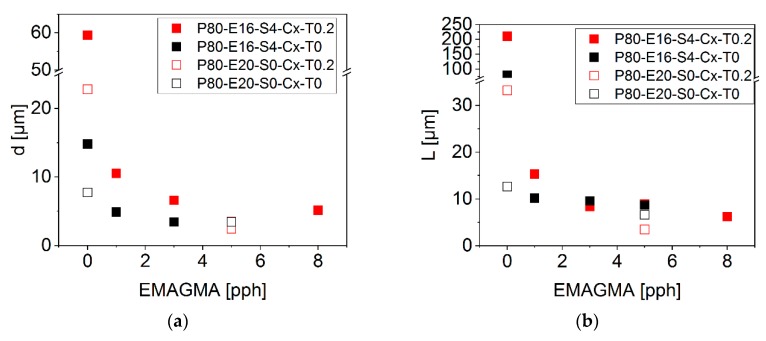
Results of scanning electron microscopy (SEM) analysis of the cryo-sliced blends. (**a**) Calculated values of particle size and (**b**) calculated values of inter particle distance.

We studied the particle size distribution of the dispersed EPDM phase in more detail by using the open source ImageJ software for calculating the particle sizes and inter particle distances according to Equations (6)–(8). The results are shown in Figure 8.

As we see, the particle size as well as the inter particle distance significantly decrease upon adding EMAGMA to the blends. Adding SBO to the blends without EMAGMA leads to an increase in particle size and inter particle distance. This may be caused by a bigger difference in viscosity since SBO acts as a plasticizer for the soft phase. This effect is even more pronounced for the samples containing TBEC. Adding EMAGMA diminishes the difference between the samples with and without TBEC.

**Figure 9:**
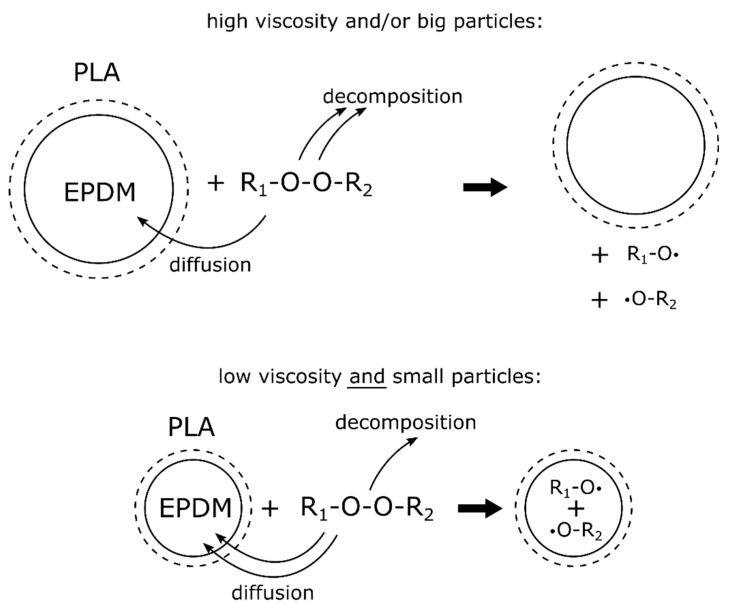
Proposed mechanism for peroxide diffusion and decomposition in the PLA–EPDM blends investigated.

According to these results, we deduce the following theoretical model: During the reactive extrusion process, the peroxide decomposition starts inside the blend matrix, i.e., PLA. In case the initial particles of the soft phase are big, which is determined by the content of EMAGMA, and the viscosity of the soft phase, which is determined by the content of SBO, is too high, the diffusion of the peroxide into the soft phase is hindered The interfacial area is small due to the large EPDM particles and, additionally, the EPDM’s viscosity is too high for a fast diffusion of the peroxide. Hence, the peroxide decomposition and following radical reactions mainly take place in the PLA phase. This leads to an even higher matrix viscosity and consequently to a bigger difference in viscosity between matrix and dispersed phase. The resulting decrease in the Weber number of the system leads to bigger EPDM particles as can be seen in the results. Adding SBO to the system decreases the viscosity of the EPDM phase. Without EMAGMA, the initial particle size of the EPDM phase is not small enough for a fast diffusion. This leads to an even higher viscosity ratio and, thus, to bigger EPDM particles what can be seen in the results. Adding EMAGMA to the blends decreases the initial size of the soft phase and thus increases the interfacial area where diffusion of the peroxide between the phases can take place. Thus, the particles of the soft phase in blends with and without TBEC tend to be of smaller and more similar sizes with increasing content of EMAGMA. The proposed model is illustrated in Figure 9.

### 3.3. Mechanical Properties

**Figure 10:**
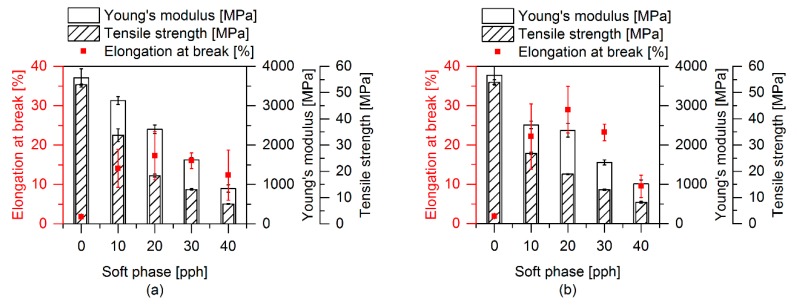
Tensile properties with varying ratios of soft phase. All samples contain 5 pph EMAGMA and 20 wt % SBO inside the soft phase. (**a**) Samples without TBEC and (**b**) samples containing 1 phr TBEC (with reference to soft phase).

Tensile and impact tests were performed to investigate the effect of the different morphological and interfacial properties on the mechanical properties of the prepared polymer blends. Figure 10 shows the influence of the soft phase on the tensile properties. All samples contain EMAGMA and the soft phase includes 20 wt % of SBO. It can be observed that the Young’s modulus as well as the tensile strength decrease with increasing content of soft phase independent of TBEC content in the blends. This can be explained by an additive mechanism of these mechanical properties based on the volumetric content of the phases in the blend, which is not influenced by the addition of peroxide. Basically, there is no significant influence of the peroxide addition on the Young’s modulus and the tensile strength of these materials. Only the sample containing 10 pph of soft phase and 1 phr TBEC shows a lower modulus of 2.5 GPa and a tensile strength of 26.9 MPa compared to the sample without TBEC with 3.1 GPa and 33.8 MPa, respectively. The elongation at break exhibits a different behavior compared to the course of the Young’s modulus and the tensile strength with increasing soft phase content. First, the elongation at break increases up to 20 pph soft phase content, but for higher soft phase content it decreases. The addition of peroxide and the induced radical reactions at the interface and/or in the soft phase increase the value of the elongation at break compared to the corresponding samples without the use of peroxide. This indicates an improved stress transfer between the matrix and the dispersed soft phase during the tensile test.

**Figure 11:**
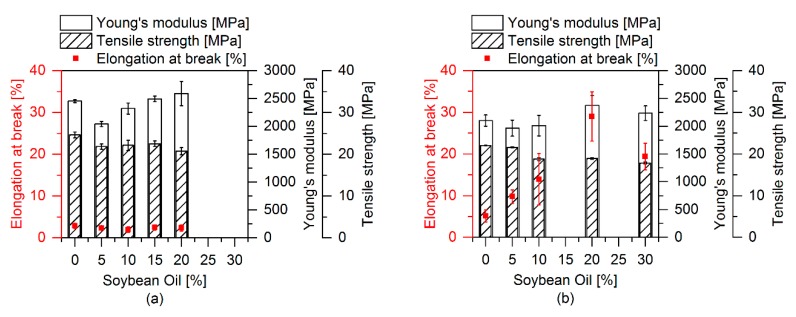
Tensile properties with varying contents of SBO inside the soft phase. Each sample contains 20 pph of soft phase. (**a**) Samples without EMAGMA and TBEC and (**b**) samples containing 5 pph EMAGMA and 0.2 pph TBEC.

Figure 11 depicts the influence of the SBO content on the tensile properties. In samples without EMAGMA and TBEC it interestingly affects the Young’s modulus, which first decreases upon adding 5 wt % of SBO to the soft phase and then increases with higher SBO contents. The addition of SBO does not influence tensile strength and elongation at break. When adding 5 pph EMAGMA and 1 phr TBEC, tensile strength and Young’s modulus show a similar behavior as in the samples without these additives. However, the system behaves different regarding the elongation at break, which firstly increases with an increasing content of SBO of up to 20 wt % and then decreases for 30 wt % SBO in the soft phase. Two different mechanisms that are directly connected to each other are responsible for these results. First, the addition of SBO leads to a reduction of the EPDM’s viscosity influencing the viscosity ratio and thus the droplet breakup as described by the Weber number (cf. previous chapter). This can even lead to a system in which the viscosity of EPDM is much lower than that of PLA and thus droplet breakup is hindered. During dynamic vulcanization, the viscosity of the soft phase increases again due to the formation of crosslinks. This increases the viscosity ratio, which favors droplet breakup of the soft phase leading to smaller particles and a higher elongation at break. Secondly, the unsaturated fatty acid chains in the SBO can also react with the radicals formed by peroxide decomposition. This reaction can lead to two different effects. On the one hand, SBO can contribute to the EPDM crosslinking as a small multifunctional crosslinking agent. On the other hand, SBO can decrease the crosslinking efficiency of EPDM by consuming free radicals without reacting with the EPDM phase. Regarding the elongation at break, the results indicate that an optimum SBO content is at about 20 wt % inside the soft phase.

**Figure 12:**
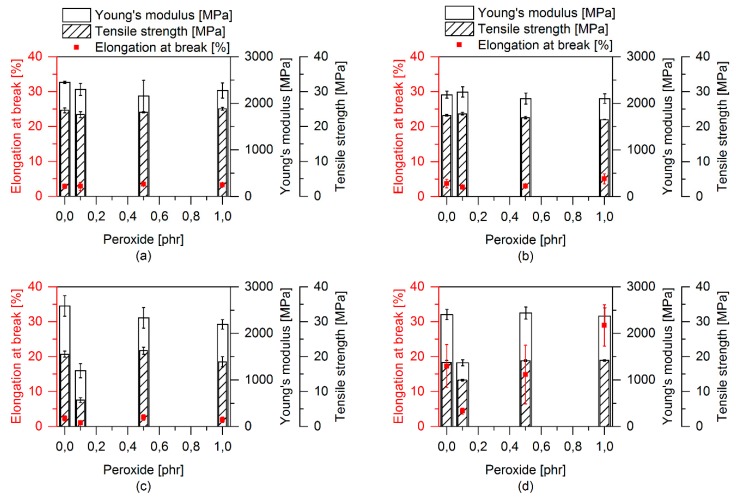
Tensile properties with varying TBEC content. Each sample contains 20 pph of soft phase. (**a**) Samples without SBO and EMAGMA, (**b**) samples containing 5 pph EMAGMA, (**c**) samples containing 20 wt % SBO inside the soft phase and (**d**) samples containing 5 pph EMAGMA and 20 wt % SBO inside the soft phase.

The effect of the peroxide addition, alone and in combination with SBO and EMAGMA as well as in combination with TBEC, EMAGMA, and SBO, is shown in Figure 12. The PLA–EPDM blend with TBEC alone and the blends with EMAGMA and TBEC do not show any significant changes in tensile properties. The blend containing 20 wt % SBO inside the soft phase is the only one to exhibit a decrease in Young’s modulus and tensile strength with a TBEC content of 0.1 phr. The other blends with SBO and higher TBEC contents do not show any significant changes. When combining all additives, i.e., EMAGMA, SBO, and TBEC, a substantial change in the tensile properties, especially regarding the increase in elongation at break in the blend containing 1 phr TBEC is observed.

**Figure 13:**
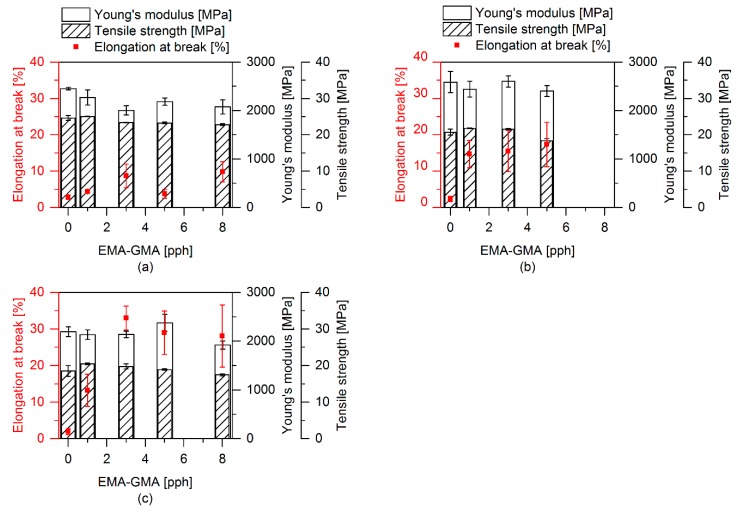
Tensile properties with varying ratios of EMAGMA. Each sample contains 20 pph of soft phase. (**a**) Samples without SBO and TBEC, (**b**) samples with 20 wt % SBO inside the soft phase, and (**c**) samples with 20 wt % SBO inside the soft phase and 0.2 pph TBEC.

The influence of the content of EMAGMA is depicted in Figure 13. The blends without SBO and TBEC show a slight increase in elongation at break, but no significant change in the other tensile properties. When adding SBO to the blends, the tensile strength decreases from about 25 MPa without SBO to 20 MPa and the Young’s modulus does not change considerably. The addition of EMAGMA leads to a substantial increase in elongation at break from about 2% to nearly 20%. When adding both, SBO and TBEC, to the blend, the increase in elongation at break is even more impressive and reaches values of more than 30%, which is 15-fold higher than for PLA and the pure blend of PLA and EPDM. These results clearly indicate the interrelationship of all the additives and thus corroborate the proposed mechanism of peroxide diffusion and decomposition. On the other hand, our results also show that small particle sizes and inter particle distances alone are not sufficient to increase the mechanical properties of PLA–EPDM blends. The materials without SBO and TBEC also comprise of small EPDM particle sizes and inter particle distances. However, only the combination of EMAGMA, SBO, and TBEC leads to an enhancement of the mechanical properties, especially regarding the elongation at break. This indicates that interfacial adhesion together with the properties of the different polymeric phases of the TPVs plays a crucial role for their mechanical properties.

**Figure 14:**
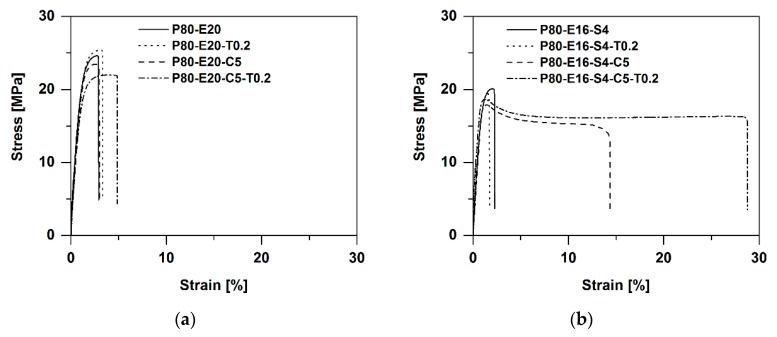
Tensile tests of the corresponding samples. (**a**) Samples without SBO and (**b**) samples with SBO.

Figure 14 summarizes the results of the mechanical tests for tensile properties and depicts the influence and importance of the combination of EPDM with SBO, EMAGMA, and TBEC. In Table 4 the characteristic values of these samples are listed.

**Figure 15:**
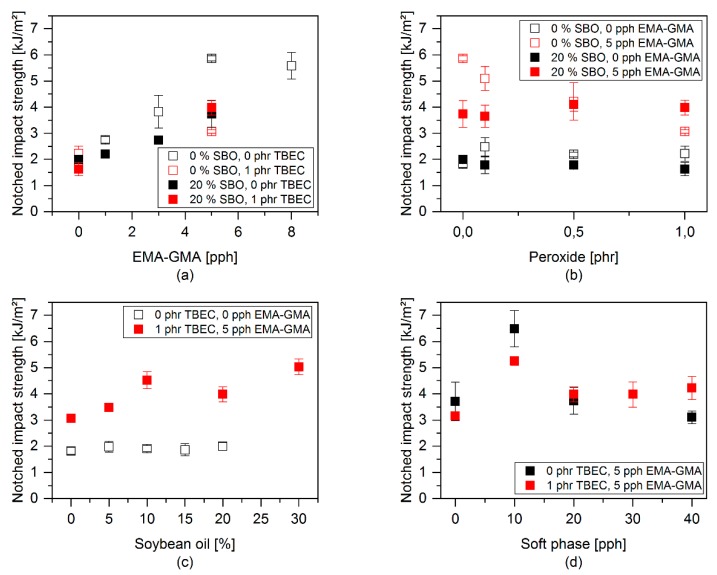
Notched impact properties with variation of different components: (**a**) Variation of EMAGMA content, (**b**) variation of TBEC content, (**c**) variation of SBO content, and (**d**) variation of soft phase content.

The impact properties with varying ratios of the additives are shown in Figure 15. The amount of EMAGMA exhibits the most significant influence on the impact values, which increase from around 2 kJ/m^2^ to around 6 kJ/m^2^ with increasing content of EMAGMA. The addition of peroxide does not influence the impact strength for the samples with SBO and the sample without SBO and EMAGMA. In the blend containing EMAGMA without SBO, an increasing content of peroxide leads to a decrease in the notched impact strength. This is also in accordance with the proposed model, since the peroxide mainly reacts in the PLA phase in this case. This leads to a more brittle matrix and, thus, decreases the impact strength. For the other blends, i.e., without SBO and EMAGMA as well as with SBO and without EMAGMA, respectively, no decrease can be observed, because the impact properties were not enhanced at all. The blend containing 20 wt % SBO and 5 pph EMAGMA does not show any influence of the content of peroxide on the impact properties.

**Figure 16:**
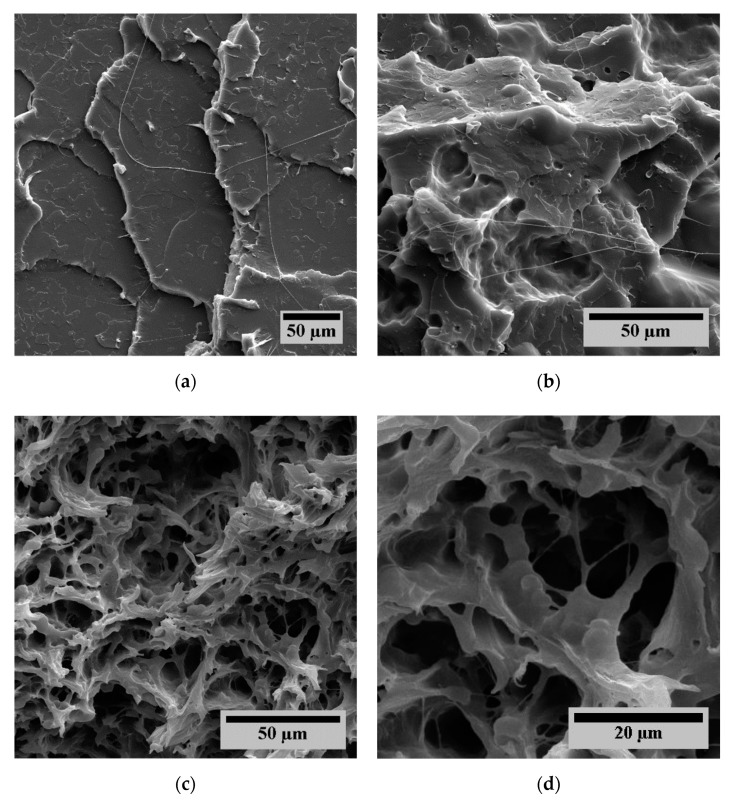
Fracture surfaces of tensile tests. (**a**) P100, (**b**) P80-E20, (**c**) P80-E16-S4-C5-T0.2, and (**d**) P80-E16-S4-C5-T0.2 with a higher magnification.

To further analyze the influence of the different additives, scanning electron microscopy (SEM) images were taken from tensile fracture surfaces, which are shown in Figure 16. Pure PLA shows brittle fracture behavior without any forms of crazing but a smooth surface with clear fracture edges (Figure 16a). Adding EPDM to the matrix does not enhance the mechanical properties, as we know from the previously shown results. The fracture surface also shows brittle fracture behavior in the PLA domains. Because EPDM is not bound to the PLA matrix, the EPDM particles can easily be torn apart from the matrix at the fracture surface. This is indicated by voids on the surface (Figure 16b). The sample containing EPDM, SBO, EMAGMA, and TBEC shows a very different behavior. The material shows a more ductile fracture behavior. This is indicated by the presence of large plastic deformation structures of the polymer phases instead of exhibiting plane fracture surfaces (Figure 16c). It can also be seen in this sample that the adhesion between the polymeric phases is much better compared to the other samples. The EPDM phase seems to be connected to the PLA phase which is indicated by the deformation of the EPDM instead of getting separated from the PLA phase (Figure 16d).

### 3.4. Thermal Properties

**Figure 17:**
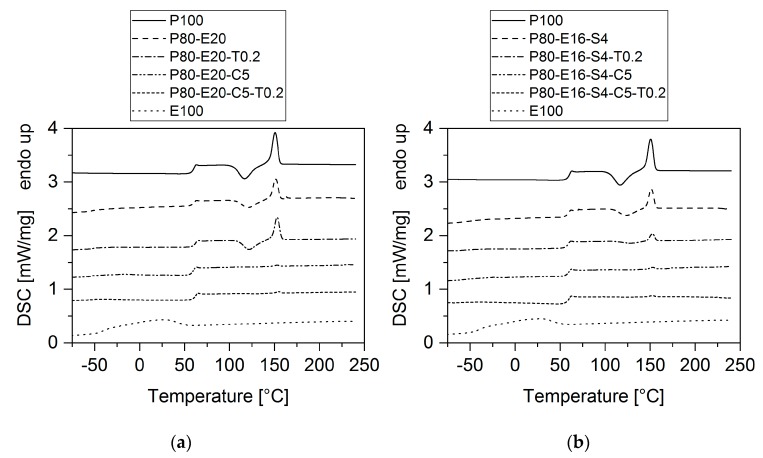
Second heating run of the differential scanning calorimetry (DSC) measurements of the corresponding samples. (**a**) Samples without SBO and (**b**) samples with SBO. The curves have been shifted for better comparability.

The dispersion of EPDM and EMAGMA inside the PLA matrix affects its thermal behavior. To determine these effects, DSC measurements were performed. Figure 17 shows the second heating run of the corresponding samples.

The addition of EPDM to PLA does not have a big influence on the thermal properties. Immiscibility of the polymer phases is visible from two separate glass transitions that are nearly the same value as for the pure polymers. Cold crystallization of the PLA is almost not affected by the addition of EPDM. For the sample containing TBEC without SBO, nearly no changes in thermal properties can be observed, too. In the sample with SBO and TBEC, however, the enthalpy of the PLA cold crystallization decreases significantly. EMAGMA exhibits the most striking influence on cold crystallization of PLA: nearly no cold crystallization is visible at all. Wang et al. have investigated the crystallization kinetic of PLA blends with a thermoplastic polyester elastomer (TPEE) compatibilized with a small multifunctional epoxide [47]. They have seen that the crystallization half time increases significantly with increasing the content of the multifunctional epoxide and assigned these results to the reaction between the epoxide and the PLA. This is in accordance to our results.

## 4. Conclusions

In this work, we investigated the compatibilization of a TPV consisting of the bio-polyester PLA as the matrix material and a partly bio-based EPDM as the elastic and dispersed soft phase. To enhance the compatibility between the two phases we used EMAGMA as a compatibilizer, SBO to change the EPDM’s viscosity and reactivity as well as a peroxide, i.e., TBEC, to induce dynamic vulcanization of the blend. We observed that EMAGMA serves as a physical compatibilizer due to the reduction of the interfacial tension between PLA and EPDM. Thus, the addition of EMAGMA reduces the particle size of the EPDM phase inside the PLA matrix and consequently enhances the mechanical properties, especially impact strength. We also found that the dynamic vulcanization with TBEC depends on the particle size of the soft phase as well as on its viscosity. We propose that decreasing particle size and viscosity influences the decomposition and diffusion of the peroxide in such a way that leads to crosslinking reactions mainly inside the soft phase. The mechanical properties of the TPVs corroborate this model. We noticed significant changes especially in elongation at break when combining all different additives and their effects.

In summary, the elongation at break increased from 2.8% for the pure blend of PLA and EPDM to 29 ± 5.9% for the compatibilized TPV. Charpy notched impact strength increased from 2.4 kJ/m^2^ for PLA to 4.0 kJ/m^2^ for the compatibilized TPV and to 5.9 kJ/m^2^ for the compatibilized blend. Dynamic vulcanization did not influence the thermal properties of the blend significantly with the exception of the addition of EMAGMA, which leads to diminishing cold crystallization of the PLA.

In the future, we are going to investigate the influence of the combination of EPDMs viscosity and peroxide diffusion and decomposition in more detail by using a non-reactive hydrogenated SBO and determination of the corresponding viscosities to further corroborate or adjust the proposed model. Furthermore, we are going to examine the possible reaction between EMAGMA as compatibilizer with PLA to enhance the compatibility between the different polymeric phases further. To investigate the compatibilization between the different polymer phases in more detail we are also going to perform dynamic rheological measurements. The results will be presented in a following paper. With this knowledge, it will be possible to tune the properties of such TPVs for achieving tailor-made solutions for different applications.

## Figures and Tables

**Table 1:** Test Liquids and Corresponding Surface Tension Components.

Test Liquid	$\sigma_{l}^{d}\;[\mathrm{mN}/m]$	$\sigma_{l}^{p}\;[\mathrm{mN}/m]$	$\sigma_{l}^{total}\;[\mathrm{mN}/m]$	Reference
Water	21.8	51.0	72.8	[30,31]
Dimethyl sulfoxide (DMSO)	36.0	8.0	44.0	[30,31]
Ethylene glycol	29.0	19.0	48.0	[30,31]
Thiodiglycol	38.4	15.6	54.0	[32]

**Table 2:** Contact Angles and Calculated Surface Free Energy (SFE) of Poly(lactic acid) (PLA), Ethylene-Propylene-Diene-Rubber (EPDM) and poly(Ethylene-Co-Methyl Acrylate-Co-Glycidyl Methacrylate) (EMAGMA).

Sample	Contact Angle [°]				SFE [mN/m]		
	Water	Ethylene Glycol	Thiodiglycol	DMSO	$\sigma_{t}$	$\sigma_{d}$	$\sigma_{p}$
PLA	63.4 ± 1.8	26.2 ± 1.5	27.8 ± 1.6	n.a. *	46.0 ± 1.7	33.1 ± 1.1	13.0 ± 0.6
EPDM	84.0 ± 2.2	69.8 ± 3.0	77.4 ± 2.9	54.8 ± 2.4	23.8 ± 2.2	14.4 ± 1.2	9.4 ± 1.0
EMAGMA	83.5 ± 1.6	68.9 ± 2.3	64.5 ± 2.3	52.4 ± 1.5	26.5 ± 1.7	18.8 ± 1.0	7.7 ± 0.7

* not applicable because dimethyl sulfoxide (DMSO) is a solvent for PLA.

**Table 3:** Calculated Interfacial Tensions (IFT), Spreading Coefficients, and Wetting Coefficients of the Used Materials.

Interface	IFT Harmonic Mean [mN/m]	IFT Geometric Mean [mN/m]
PLA–EPDM	7.9	4.1
PLA–EMAGMA	5.3	2.7
EPDM–EMAGMA	0.7	0.4
Spreading coefficient	1.9	1.0
Wetting coefficient	−0.6	−0.6

**Table 4:** Mechanical Properties of the Corresponding Samples from Tensile and Charpy Impact Tests.

Sample	Elongation at Break [%]	Tensile Strength [MPa]	Young’s Modulus [GPa]	Charpy Notched Impact Strength [kJ/m^2^]
P100	2.0 ± 0.2	65.9 ± 2.3	4.9 ± 0.3	2.4 ± 0.5
E100	1130 *	4 *	4E-3 *	n.a. **
P80-E20	2.8 ± 0.6	24.6 ± 0.7	2.5 ± 0.1	1.8 ± 0.1
P80-E20-T0.2	3.3 ± 0.2	25.1 ± 0.4	2.3 ± 0.2	2.2 ± 0.3
P80-E20-C5	3.7 ± 1.2	23.2 ± 0.3	2.2 ± 0.1	5.9 ± 0.1
P80-E20-C5-T0.2	5.1 ± 1.4	22.0 ± 0.1	2.1 ± 0.1	3.1 ± 0.1
P80-E16-S4	2.3 ± 0.8	20.7 ± 0.8	2.6 ± 0.2	2.0 ± 0.2
P80-E16-S4-T0.2	1.9 ± 0.8	18.5 ± 1.4	2.2 ± 0.1	1.6 ± 0.2
P80-E16-S4-C5	17.3 ± 6.1	18.4 ± 0.6	2.4 ± 0.1	3.7 ± 0.5
P80-E16-S4-C5-T0.2	29.0 ± 5.9	18.9 ± 0.2	2.4 ± 0.2	4.0 ± 0.3

* only one single measurement; ** not applicable.
